# Association between obsessive-compulsive disorder and the risk of schizophrenia using the Korean National Health Insurance Service-National Sample Cohort: a retrospective cohort study

**DOI:** 10.1017/S2045796023000021

**Published:** 2023-02-10

**Authors:** H. Kim, S. H. Kim, W. Jeong, Y. S. Park, J. Kim, E. C. Park, S. I. Jang

**Affiliations:** 1 Department of Preventive Medicine, Yonsei University College of Medicine, Seoul, Republic of Korea; 2 Institute of Health Services Research, Yonsei University, Seoul, Republic of Korea; 3 Department of Psychiatry, Yonsei University College of Medicine, Seoul, Republic of Korea; 4 Cancer Knowledge & Information Center, National Cancer Control Institute, National Cancer Center, Goyang, Republic of Korea; 5 Department of Public Health, Graduate School, Yonsei University, Seoul, Republic of Korea; 6 Department of Preventive Medicine, Eulji University School of Medicine, Daejeon, Republic of Korea

**Keywords:** Obsessive-compulsive disorder, risk factors, schizophrenia, survival analysis

## Abstract

**Aims:**

Obsessive-compulsive disorder (OCD) and schizophrenia are often reported as co-morbid conditions. However, the evidence of an association between OCD and the risk of schizophrenia is limited. This study investigated the risk of schizophrenia in patients newly diagnosed with OCD using a nationally representative sample cohort in South Korea.

**Methods:**

Data were obtained from the 2002–2013 Korean National Health Insurance Service-National Sample Cohort of the National Health Insurance Service. Using propensity score matching, 2509 patients with OCD and a control group of 7527 patients were included in the analysis. Chi-squared tests were used to investigate and compare the general characteristics of the study population. The risk of schizophrenia was analysed using the Cox proportional hazard model.

**Results:**

The incidence rate was 45.79/10 000 person-year for patients with OCD and 4.19/10 000 person-year for patients without OCD. Patients with OCD had a higher risk of schizophrenia compared to the control group after adjusting for covariates (hazard ratio = 10.46, 95% confidence interval = 6.07–18.00).

**Conclusions:**

This study identified an association between the diagnosis of OCD and the risk of schizophrenia in a South Korean national representative cohort. Further research using a prospective design to clarify the causality of OCD in schizophrenia in a controlled environment should be conducted to validate these findings.

## Introduction

Schizophrenia and obsessive-compulsive disorder (OCD) are two distinct chronic mental disorders that deteriorate the quality of life of patients and their families. The prevalence of schizophrenia ranges from 0.25 to 0.75% (Saha *et al*., 2005; McGrath *et al*., 2008; Moreno-Küstner *et al*., 2018). Schizophrenia induces excess mortality that reduces life expectancy by almost 20 years (Laursen *et al*., 2014). The lifetime prevalence of OCD among US adults is 2.3% (Ruscio *et al*., 2010) and ranges from 0.6 to 0.8% in the South Korean population (Cho *et al*., 2004; Hong *et al*., 2009). Previous studies showed that OCD is associated with an increased mortality risk (mortality rate ratio = 2.00) compared to the general population (Meier *et al*., 2016). A large sample cohort study suggested that patients with OCD had a 9.83 times higher risk of death by suicide compared to matched controls (Fernández de la Cruz *et al*., 2017). Although these two conditions are regarded as different disorders, they share some clinical characteristics, such as the similarity between obsessions and delusions. They show significant comorbidity, which may lead to initial misdiagnosis of OCD for schizophrenia patients (Rasmussen *et al*., 2020). This similarity has led many researchers to focus on their association (Bottas *et al*., 2005; Schirmbeck and Zink, 2013; Sharma and Reddy, 2019).

Obsessive-compulsive symptoms (OCSs) may occur after the administration of atypical antipsychotics such as clozapine, which suggests that co-morbid OCD in schizophrenia patients could be a result of the side effects of this drug (Bleakley *et al*., 2011; Kim *et al*., 2019). However, OCSs or OCD may occur at any point during schizophrenia (Poyurovsky *et al*., 2004). In addition, co-morbid OCSs were substantially observed in adolescent schizophrenia patients suggesting that OCD is not just a result of side effects of the drug. The disease may be associated with schizophrenia (Nechmad *et al*., 2003). Moreover, schizophrenic patients with OCSs showed severe psychotic symptoms, earlier onset, longer hospital stay and poorer prognosis, which suggests that schizophrenia with OCSs has different characteristics compared to schizophrenia without OCSs (Berman *et al*., 1995; Cunill *et al*., 2009). On the other hand, the prodromal characteristics of patients with schizophrenia include the obsessive-compulsive phenomenon, and they may thus be diagnosed with OCD before diagnosing schizophrenia (Niendam *et al*., 2009; George *et al*., 2017). These previous studies suggest an association between the development of OCD and schizophrenia. Therefore, it is necessary to investigate the association between OCD and schizophrenia.

Previous studies have suggested that the comorbidity of OCD and schizophrenia was greater than 10%, suggesting that this is not related to chance alone (Swets *et al*., 2014). One meta-analysis that focused on the temporal sequence of schizophrenia and co-morbid OCD suggested that the onset of OCD precedes schizophrenia (Devulapalli *et al*., 2008). Only a few studies have investigated the risk of schizophrenia in patients diagnosed with OCD. Cederlöf *et al*. reported that patients with OCD had a 12-fold increased risk of a co-morbid diagnosis of schizophrenia (Cederlöf *et al*., 2015) in a Swedish cohort. Meier *et al*. reported that the incidence rate ratio of schizophrenia was 6.90 among patients with OCD, compared to controls who had not been diagnosed as having OCD in Danish registers (Meier *et al*., 2014). Moreover, the offspring of OCD patients had an increased risk of schizophrenia (Incidence Rate Ratio (IRR) = 3.10). A Taiwanese study also concluded that an OCD group had a higher risk of schizophrenia than a non-OCD group (hazard ratio [HR] = 30.29), and OCD patients co-morbid with autistic disorder showed an increased risk for schizophrenia (HR = 4.63) (Cheng *et al*., 2019). To further strengthen the association between OCD and the consequences of schizophrenia, nationally representative studies in different countries should be conducted.

This study aimed to investigate the association between a history of OCD and the risk of schizophrenia in a Korean nationally representative cohort using nationwide claims data after adjusting for covariates.

## Method

### Study population and data

The data analysed were acquired from the Korean National Health Insurance Service-National Sample Cohort (NHIS-NSC) of the National Health Insurance Service (NHIS) between 2002 and 2013. The Korean NHIS provides researchers with all data on claims collected under the NHIS for academic investigation and policy making. The NHIS-NSC data include all medical claims from 1 025 340 individuals, accounting for 2% of the South Korean population by random sampling. The NHIS-NSC database provides information on socioeconomic status and clinically determined International Classification of Diseases, 10th revision (ICD-10) codes. The NHIS-NSC data were de-identified. Thus, the institutional review board waived the need for informed consent. All patients in the cohort were followed up unless they were excluded because of death or migration.

Patients who sought treatment for OCD (F42) or schizophrenia, schizotypal and delusional disorder (F2X) in 2002 were excluded as this study aimed to investigate patients with newly developed OCD and schizophrenia. The database started in 2002. Thus, patients diagnosed in 2002 could be diagnosed initially before the study period. We excluded patients from the analysis with a difference of less than 1 year between the diagnosis date of OCD and schizophrenia to reduce the possibility of reverse causality and initial misdiagnosis of the two diseases. The sensitivity analysis for the period between the date of diagnosis is presented in online Supplementary Table 1. The OCD patients were included in the study population when they first received the diagnostic code for OCD. They were followed up until they died or received a diagnostic code for schizophrenia or the end of the study period (2013.12.31). After these exclusions, 2509 patients were included in the OCD group. A control group was included based on 1 : 3 propensity score matching using logistic regression to calculate the probability with covariates of sex, age group, insurance, index year, income status and OneToManyMTCH macro run in SAS (Parsons, 2004). The *c*-statistics of the propensity model was 0.601.

### Study variables and covariates

Patients were considered to have OCD based on the diagnostic ICD-10 code F42 at the first visit. Patients with ICD-10 code F20 were considered to have schizophrenia. Baseline demographic information, including age, sex, health insurance status, residential region, disability, income status, and comorbidity, including the Charlson comorbidity index (CCI), were included in the regression model as covariates. CCI score is an index for assessing the patients' comorbidities for use in longitudinal studies using administrative data. The score was calculated by weighting 1–6 scores for 19 co-morbid diseases (Sundararajan *et al*., 2004). The weighted score was assigned to each of the 17 comorbidities based on the relative 1-year risk of mortality. Participants were divided into three groups according to their CCI scores: 0–2, 3–4 and ⩾5. Age was categorised into seven groups (0–9, 10–19, 20–29, 30–39, 40–49, 50–59 and ⩾60). The residential region contains three categories concerning population density: metropolitan (Seoul, Gyeonggi-do), city (Busan, Daegu, Incheon, Gwangju, Daejeon, Ulsan) and rural (Gangwon-do, Chungcheong-do, Jeolla-do, Gyeongsang-do, Jeju-do). Income status was divided into three groups by their health insurance premium, which is in proportion to their income. Having a disability was defined as someone who had registered as a person with a disability to the government to receive benefits. Both mental and physical disabilities were included.

### Statistical analysis

Chi-squared tests were used to investigate and compare the general characteristics of the study population. A Cox proportional hazard model was generated to examine the association between OCD and the risk of schizophrenia. The covariates were included in the analyses. Subgroup analyses stratified by covariates were performed to investigate the combined effects of OCD and the other covariates on schizophrenia. The results are presented as HRs and confidence intervals (CIs) to compare the risk of schizophrenia among the groups. All analyses were conducted using SAS software version 9.4 (SAS Institute, Cary, North Carolina, USA); *p*-values <0.05 were considered statistically significant.

### Ethical considerations

The database used in this study is from routinely collected administrative and claims data. As the NHIS-NSC data did not contain identifying information, the study was approved by the Institutional Review Board of Yonsei University's Health System (4-2021-0155). The study adhered to the principles of the Declaration of Helsinki.

## Results

The general characteristics of the study population are summarised in Table 1. A total of 10 036 patients were included in the analysis, and 0.8% were diagnosed with schizophrenia. The mean follow-up time was 5.31 years for patients with OCD and 5.39 years for patients without OCD. The chi-squared test showed a significant difference in the prevalence of schizophrenia between groups divided by the diagnosis of OCD. Age, social security, region, disability status and history of a pervasive developmental disorder and intellectual disability had significant effects on the risk of schizophrenia in univariate analysis. Sex, income and CCI were not associated with schizophrenia during the study period.

**Table 1.** General characteristics of the study population and the results of chi-squared test with the risk of schizophrenia

Variables	Total	Schizophrenia		*p* value
		Yes	No	
Total	10 036 (100.0)	78 (0.8)	9958 (99.2)	
Obsessive compulsive disorder				<0.0001
Yes	2509 (25.0)	61 (2.4)	2448 (97.6)	
No	7527 (75.0)	17 (0.2)	7510 (99.8)	
Sex				0.7026
Male	5188 (51.7)	42 (0.8)	5146 (99.2)	
Female	4848 (48.3)	36 (0.7)	4812 (99.3)	
Age				0.0534
0–9	1344 (13.4)	6 (0.4)	1338 (99.6)	
10–19	1972 (19.6)	22 (1.1)	1950 (98.9)	
20–29	1868 (18.6)	16 (0.9)	1852 (99.1)	
30–39	1700 (16.9)	18 (1.1)	1682 (98.9)	
40–49	1380 (13.8)	6 (0.4)	1374 (99.6)	
50–59	876 (8.7)	2 (0.2)	874 (99.8)	
⩾60	896 (8.9)	8 (0.9)	888 (99.1)	
Social security				0.017
Health insurance	9868 (98.3)	74 (0.7)	9794 (99.3)	
Medical aid	168 (1.7)	4 (2.4)	164 (97.6)	
Region				0.4381
Metropolitan	4303 (42.9)	37 (0.9)	4266 (99.1)	
City	2838 (28.3)	17 (0.6)	2821 (99.4)	
Rural	2895 (28.8)	24 (0.8)	2871 (99.2)	
Disability				0.0016
Yes	233 (2.3)	6 (2.6)	227 (97.4)	
No	9803 (97.7)	72 (0.7)	9731 (99.3)	
Income				0.1664
Low	1176 (11.7)	11 (0.9)	1165 (99.1)	
Middle	4276 (42.6)	25 (0.6)	4251 (99.4)	
High	4584 (45.7)	42 (0.9)	4542 (99.1)	
CCI				0.0220
0–2	6448 (64.2)	39 (0.6)	6409 (99.4)	
3–4	2580 (25.7)	30 (1.2)	2550 (98.8)	
⩾5	1008 (10.0)	9 (0.9)	999 (99.1)	

Variables are presented as numbers and percentages.

Table 2 shows the results of the Cox proportional hazard regression analysis for the association between OCD and the risk of schizophrenia after adjusting for the covariates mentioned in Table 1. The incidence rate was 45.79/10 000 person-year for patients with OCD and 4.19/10 000 person-year for patients without OCD. Individuals with OCD had a higher risk of schizophrenia after adjusting for covariates (HR = 10.46, CI = 6.07–18.00).

**Table 2.** Results of the Cox proportional hazard regression analysis on the association between obsessive compulsive disorder and the risk of schizophrenia

	Risk of schizophrenia	
Variables	HR	95% CI
Obsessive compulsive disorder		
Yes	10.46	6.07–18.00
No	1.00	
Gender		
Male	1.00	
Female	0.94	0.59–1.48
Age		
0–9	0.81	0.31–2.09
10–19	1.27	0.65–2.45
20–29	1.00	
30–39	1.04	0.53–2.04
40–49	0.37	0.14–0.96
50–59	0.18	0.04–0.81
⩾60	0.62	0.24–1.61
Social security		
Health insurance	1.00	
Medical aid	3.89	1.05–14.41
Region		
Metropolitan	1.00	
City	0.61	0.34–1.08
Rural	1.01	0.60–1.70
Disability		
Yes	3.27	1.30–8.21
No	1.00	
Income		
Low	1.00	
Middle	0.85	0.37–1.97
High	1.31	0.58–2.93
CCI		
0–2	1.00	
3–4	1.67	1.01–2.77
⩾5	1.48	0.63–3.48

Independent subgroup analyses were conducted to assess the combined effects of OCD history and other sociodemographic variables on the risk of schizophrenia, as shown in Table 3. When stratified by age, patients in their 20s showed a much higher risk of schizophrenia than older patients. Finally, patients with lower CCI scores had a significantly higher risk of schizophrenia (HR = 16.86, CCI = 0–2; HR = 7.44, CCI = 3–4) than those of the highest-scoring group (HR = 3.91, CCI ⩾ 5).

**Table 3.** Subgroup analysis of the association between risk of schizophrenia and covariates, according to OCD history

	No OCD	OCD	
	Adjusted HR	Adjusted HR	95% CI
Sex			
Male	1.00	11.93	5.49–25.95
Female	1.00	9.14	4.25–19.64
Age			
0–9	1.00	*	*
10–19	1.00	12.50	4.15–37.66
20–29	1.00	41.28	5.40–315.70
30–39	1.00	3.69	1.42–9.55
40–49	1.00	7.78	1.36–44.61
50–59	1.00	*	*
⩾60	1.00	6.12	6.03–18.46
Social security			
Health insurance	1.00	10.55	6.03–18.46
Medical aid	1.00	*	*
Region			
Metropolitan	1.00	8.66	4.05–18.54
City	1.00	41.09	5.42–311.60
Rural	1.00	8.06	3.29–19.76
Income			
Low	1.00	14.71	3.11–69.62
Middle	1.00	8.36	3.31–21.13
High	1.00	11.06	5.26–23.27
Disability			
Yes	1.00	*	*
No	1.00	9.42	5.43–16.35
CCI			
0–2	1.00	16.86	7.41–38.36
3–4	1.00	7.44	3.17–17.47
⩾5	1.00	3.91	0.93–16.45

* Due to sparsity of the data, HR could not be calculated in the model.

## Discussion

A history of OCD was associated with an increased risk of schizophrenia in Korea over a 12-year follow-up period. Patients with an initial diagnosis of OCD had an approximately 10.46-fold higher risk of being diagnosed with schizophrenia during the follow-up period than individuals without an OCD diagnosis. The increase in schizophrenia risk among patients with OCD in our study is generally similar to that in previous studies. A previous study reported that the incidence rate of schizophrenia was 6.9 times higher in patients with OCD compared to that in non-OCD participants (Meier *et al*., 2014), and the HR of schizophrenia may increase over time, as reported in previous studies (Cheng *et al*., 2019). Compared to the Taiwanese cohort study, our result (HR = 10.46) was lower than the adjusted risk (HR = 30.29) in that study, probably due to the washout period in our study design. We excluded patients diagnosed with schizophrenia within 1 year after the initial diagnosis of OCD, as the OCSs might have been an early manifestation of schizophrenia or the misdiagnosis.

How OCD and the development of schizophrenia have an association has not yet been clearly established. However, previous studies suggest possible explanations. Both schizophrenia and OCD are hypothesised to have a neurodevelopmental aetiology (Murray and Lewis, 1987; Huyser *et al*., 2009; Rapoport and Gogtay, 2011; Ivarsson *et al*., 2017), and our results may be the consequence of a common neurodevelopmental pathology. Some studies have attempted to identify common neurological deficits to reveal this association, but definitive evidence is still unclear (Gross-Isseroff *et al*., 2003; Bottas *et al*., 2005). Further studies are needed to determine the mechanisms underlying the association between the two disorders.

The high association between OCD and schizophrenia might be due to the similarity of the symptoms, which led to the initial misdiagnosis for patients. It is difficult to differentiate obsessions with low insight and delusions; thus the Diagnostic and Statistical Manual of Mental Disorders fifth edition text revision (DSM-5-TR) suggests the ‘delusional beliefs’ specifier for OCD (American Psychiatric Association, 2022; Rasmussen and Parnas, 2022). A previous study suggested that 29% fulfilled the criteria of schizophrenia among OCD patients (Rasmussen *et al*., 2020). Moreover, almost 30% of schizophrenia patients show OCSs (Tezenas du Montcel *et al*., 2019). Thus, schizophrenia patients who present OCSs dominantly in their prodromal stage might be diagnosed with OCD, which turns to schizophrenia in their active phase. As we used retrospective insurance claims data, we could not exclude the possibility that primary OCD diagnosis is a misdiagnosis. However, we excluded the OCD patients who received the diagnosis of schizophrenia within the first follow-up year; thus, the possibility of misdiagnosis might be lowered.

The subgroup analysis showed that the lower CCI group had the highest risk of schizophrenia by OCD diagnosis (HR = 15.49 in CCI = 0–2) that decreased in the higher CCI group (HR = 7.46 in CCI = 3–4; HR = 3.83 in CCI ⩾ 5). These results are probably due to the heterogeneity of schizophrenia based on its onset age. The higher CCI group tends to be older because number of comorbid diseases are highly correlated with older age. Thus, the higher CCI group contains more late-onset schizophrenia patients than the lower CCI group. One previous study tried to differentiate between risk factors among early- and late-onset schizophrenia patients. Their results showed that physical health factors including malignance, chronic illnesses and cardiovascular disease were significant for late-onset schizophrenia but not for youth- or middle-onset schizophrenia (Chen *et al*., 2018). Our results might suggest that the association between OCD and the risk of schizophrenia might be higher in early-onset schizophrenia but lower in late-onset schizophrenia. The higher risk for schizophrenia in younger participants from the subgroup analysis also supports the idea.

This study had several limitations. First, as this was a retrospective cohort study, we could not include all the variables affecting schizophrenia development. A family history of schizophrenia, one of the strongest risk factors for developing schizophrenia, could not be included in the analysis because NHIS cohort data did not contain that information. Second, the accuracy of the diagnostic information may have been limited because of the inaccuracy of the claims diagnosis, as suggested in a previous study (Park, 2017). We investigated the primary and secondary diagnostic codes and included both in the analysis to increase diagnostic accuracy. Third, the data lacked psychometric measures; thus, we could not accurately determine the severity and types of symptoms. Fourth, among the wide range of schizophrenia spectrum disorders, we only included schizophrenia; thus, we could not find an association between OCD and other psychotic disorders. Our initial purpose of the study was to investigate the association between OCD and schizophrenia; thus, we excluded patients with codes for other psychotic disorders because other codes, such as F28 and F29, might be inserted to prescribe antipsychotics for affective disorders, delirium and sleep disorders. However, diagnosing schizophrenia is rather strict; thus, we decided to use the narrow diagnostic code of F20. Lastly, we used the insurance claims data. Therefore, only the treated population could be involved in the study. Visiting a psychiatrist and receiving treatment is still a hurdle for many people with symptoms. Thus, our findings could not capture those populations whose incidence and prognosis of psychiatric disease might differ from our study subjects (Font *et al*., 2018).

Despite these limitations, our study had several strengths. Although claims data have limitations, we used national sampling cohort data representing the general population of Korea. The results of our research can be generalised to the entire population of South Korean individuals. They may provide a background for early detection and management of schizophrenia among OCD patients, thus improving patient prognosis.

This is the first study to identify an association between a history of OCD and the risk of schizophrenia in a South Korean national representative cohort. Further research using a prospective design to clarify the causality of OCD in schizophrenia in a controlled environment should be conducted to validate these findings.

## Data Availability

The Korean National Health Insurance Service-National Sample Cohort is a public, open-access database. It is based on the health insurance claim data of all Koreans, and the sample cohort is available for public purposes and scientific research. The sample cohort data are available after acceptance of approval for use by the national health insurance service (https://nhiss.nhis.or.kr/bd/ab/bdaba000eng.do).

## Supplementary material

For supplementary material accompanying this paper visit http://doi.org/10.1017/S2045796023000021. click here to view supplementary material
