# Supplementary material for: Association between obsessive-compulsive disorder and the risk of schizophrenia using the Korean National Health Insurance Service-National Sample Cohort: a retrospective cohort study
Source: Epidemiol Psychiatr Sci. 2023 Feb 10;32:e9. doi: 10.1017/S2045796023000021 (PMC9971846; doi:10.1017/S2045796023000021)
Supplement: Supplementary file 1 [file S2045796023000021sup001.docx]

**Supplementary table 1. Sensitivity analysis of washout period between diagnosis of Schizophrenia after OCD for the association between OCD and the risk of schizophrenia**

| **Washout Period** | **Risk of schizophrenia** | | | |
| --- | --- | --- | --- | --- |
|  | **HR** | **95% CI** | | |
| **6 Months** | 13.67 | (8.05 | - | 23.21) |
| **1 Year** | 10.46 | (6.07 | - | 18.00) |
| **2 Years** | 6.82 | (3.85 | - | 12.08) |

All covariates in table 2 were included in the Cox proportional hazard model.
